# Longer‐term outcome of acute pancreatitis: 5 years follow‐up

**DOI:** 10.1002/jgh3.12679

**Published:** 2021-11-19

**Authors:** Partha Sarathi Patra, Kshaunish Das

**Affiliations:** ^1^ Divisions of Gastroenterology, School of Digestive and Liver Disease Institute of Post‐Graduate Medical Education and Research Kolkata India

**Keywords:** acute pancreatitis follow‐up, chronic pancreatitis, diabetes, long‐term complications

## Abstract

**Background and Aim:**

Following an index episode of acute pancreatitis, sometimes the inflammation subsides completely, but sometimes inflammation persists and progresses to chronic pancreatitis, which may be or may not be preceded by recurrent acute pancreatitis. Some patients may also develop diabetes mellitus. There is only limited information on the longer‐term outcome of patients with acute pancreatitis. The aim of this study was to evaluate the longer‐term consequences of acute pancreatitis in the form of the development of recurrent attacks of acute pancreatitis, chronic pancreatitis, diabetes, or pancreatic carcinoma.

**Methods:**

The index study included 122 patients who presented with their first episode of acute pancreatitis. This retrospective, cross‐sectional survey was performed 5 years after the index episode.

**Results:**

Of the 122 patients, 96 were available for follow‐up while 4 were known to have died (one from pancreatic cancer). On reassessment after 5 years, 28 of 96 patients had further episodes of pancreatitis. Fifteen patients were diagnosed as having recurrent acute pancreatitis, 13 patients were diagnosed as having chronic pancreatitis, while 17 developed new‐onset diabetes. Recurrent acute pancreatitis was more common in younger patients, while chronic pancreatitis was associated with alcohol abuse and a more severe index episode. The development of diabetes was more common with advanced age.

**Conclusions:**

In this study, a good proportion of patients progressed to chronic pancreatitis and diabetes within 5 years after surviving acute pancreatitis.

## Graphical abstract

Cross‐sectional review was done after 5 years of follow‐up of acute pancreatitis. Out of 100 patients, 15% patients developed recurrent acute pancreatitis, 13% patients developed chronic pancreatitis, and 17% patients developed diabetes.

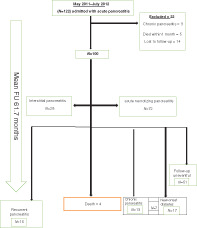

## Introduction

Acute pancreatitis (AP) is a common gastrointestinal emergency requiring hospital admission and causing a significant health‐care burden. The majority of patients recover completely after an acute attack of pancreatitis. However, some patients will go on to develop recurrent attacks of acute pancreatitis (RAP), chronic pancreatitis (CP), or endocrine insufficiency in the form of diabetes mellitus. There has been a dearth of data in this regard as studies conducted are few and far between.^1^, ^2^, ^3^ Over the years, the nomenclature and definitions of the outcomes have also undergone significant refinement. Newer studies are required to shed light on natural progression after AP and thereby helping to develop better strategies to manage the outcomes. This study evaluates the outcomes in terms of RAP, CP, diabetes, and exocrine insufficiency in a cohort admitted with AP recruited in our index study.^4^

## Materials and methods

### Study design

It is a retrospective observational study. All patients recruited in our index study with AP were followed prospectively for 1 year as per study schedule; after that, patients were advised to attend a pancreatic clinic or emergency for new‐onset pain abdomen or for any abdominal symptoms. Patients who developed diabetes were managed in the diabetic clinic of the same hospital. Investigations were done according to clinical situations. At the end of 5 years, we did a cross‐sectional survey of all recruited patients. We contacted all patients or their relatives (if the patient had died) by telephonic call and letters and did a retrospective comprehensive review of their history of any illness, clinical records, and imaging data were conducted for identification of outcomes in terms of RAP, CP, diabetes mellitus, and pancreatic malignancy.

Approval of the study was done from the Institutional Ethics Committee of the IPGMER, Kolkata. All patients (or their relatives if the patient had died) provided informed consent. This study was conducted in accordance with the Declaration of Helsinki.

### Exclusion criteria

The exclusion criteria are those mentioned in our index study.^4^ No new exclusion criteria were applied in this study.

### Diagnosis and severity of acute pancreatitis

AP diagnosis was established whenever two of the following three criteria were present: abdominal pain; elevated serum levels of amylase and/or lipase three times the upper limit; and imaging evidences of AP.^5^ Etiology of biliary cause was made when there were typical radiological or biochemical features.^5^ A diagnosis of alcohol‐related AP was made whenever the patient or patient's relative reported recent high (risky) alcohol intake or prolonged alcohol use.^5^ Other causes were made based on history (e.g. drugs, post‐endoscopic retrograde cholangiopancreatography [ERCP], or trauma). Idiopathic pancreatitis was established if other etiologies were absent. Measurement of levels of blood urea nitrogen, serum creatinine, complete blood count, packed cell volume (PCV), liver function tests, fasting blood glucose, calcium and phosphate and arterial blood gas analysis, and transabdominal ultrasound (USG) were done within 24 h of admission. Bedside Index for Severity in Acute Pancreatitis (BISAP) score at admission was noted.^6^ Type and duration of organ failure assessed according to the Marshall Score.^7^ Contrast‐enhanced computer tomography (CECT) of the abdomen was done after 5–7 days of onset of pain, and baseline CT Severity Index (CTSI) was calculated.^8^, ^9^ Severity of AP and types of fluid collection due to AP were classified and noted in accordance with the revision of the Atlanta classification and definitions by international consensus.^10^

### Follow‐up and final cross‐sectional evaluation

Of 122 patients in the index study, 4 were known to have died and 96 attended the OPD for follow‐up. Complications after AP were determined by interview and examination of medical records. Particular attention was paid to the nature, severity, and management of further episodes of abdominal pain as well as the possible development of diarrhea, weight loss, or diabetes. All patients were advised to do a fasting blood sugar and blood sugar 2 h after 75 grams of glucose ingestion except those patients who were already diagnosed with diabetes. Fasting plasma glucose value equal to or greater than 126 mg/dL and/or plasma glucose value equal to or greater than 200 mg/dL after a 2‐h glucose load confirmed on two occasions was diagnosed as diabetes.

All patients underwent transabdominal ultrasonography (USG) upper abdomen.

CECT of the upper abdomen was done if the USG abdomen showed any pancreatic ductal changes like dilatation, narrowing, calcification, or any pancreatic parenchymal calcification.

For the diagnosis of CP, the MANNHEIM diagnostic criteria were used.^11^ Definite diagnosis of CP would require any one of the following:

1. calcification in pancreas;
2. ductal abnormalities (as per Cambridge classification);
3. exocrine insufficiency is defined as pancreatic fatty diarrhea clinically reduced by enzyme supplementation; or
4. histology suggestive of CP.

Risk factors of hypercalcemia, hypertriglyceridemia were assessed in all patients of RAP but Magnetic Resonance Cholangiopancreatography (MRCP) and genetic testing were not done.

## Statistical analysis

SPSSTM (version 13 for Windows) software was used for statistical analysis. Descriptive data were reported as the mean and standard error of mean (SEM) for continuous variables, while percentages were calculated for categorical variables. We obtained Lambda and Mann–Whitney's *U* for continuous variables, Fisher's exact test for categorical variables, and univariate analysis to identify baseline predictors for development for RAP, CP, and new‐onset diabetes. A two‐tailed *P*‐value <0.05 was taken as significant.

## Results

**Figure 1:**
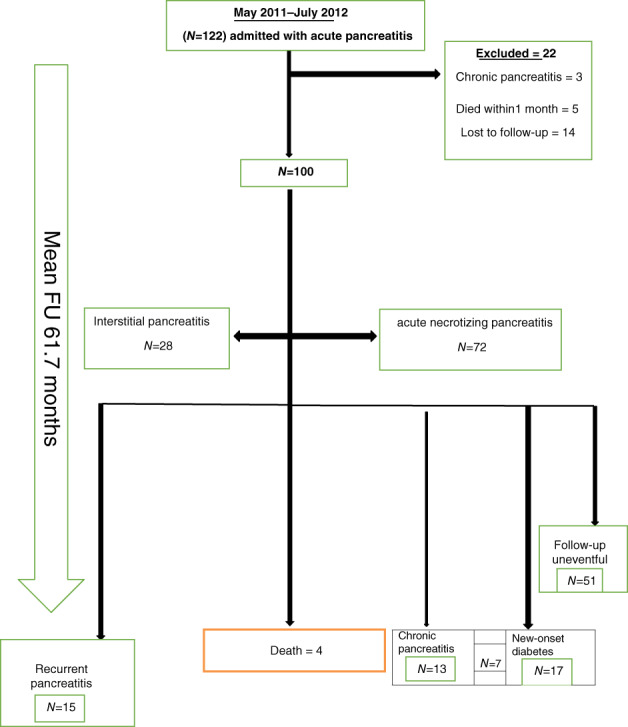
Hundred patients with acute pancreatitis (72% necrotizing acute pancreatitis) were followed up for approximately 5 years. At the end of the follow‐up, a cross‐sectional evaluation was done. On evaluation, it was found that 15% of patients had recurrent acute pancreatitis, 13% of patients developed chronic pancreatitis, and 17% of patients developed new‐onset diabetes at the end of follow‐up. Four patients died within the follow‐up time. A total of 51% of patients had uneventful follow‐up.

Of 100 patients whose outcome was known, 64 were male and 36 were female with a mean age of 42 years (range 14–88 years) at the time of the index episode of pancreatitis (Fig. 1, flowchart of outcomes of patients during or at the end of follow‐up). The etiology of index AP was alcohol in 21, biliary in 31, idiopathic in 35, post‐ERCP in 11, ascariasis‐related in 4, post‐traumatic in 3, and drug‐induced in 1 (Table 1). Of the 98 patients who had a CT scan between 5 and 7 days of onset of index AP, 72 (73.5%) had acute necrotizing pancreatitis (ANP) during index AP while an acute fluid collection (AFC) was present in 83 (83%). In 38 (46%) of these 83 patients, a pseudocyst or walled‐off‐pancreatic‐necrosis (WOPN) had developed after 4 weeks. The index episode of AP was mild, moderate, and severe in 59, 23, and 18 patients, respectively, according to the Revised Atlanta Classification.

**Table 1:** Most common etiology

Study	Country	Most common etiology
Lankisch *et al*.^3^	Germany	Biliary (42%)
Angelini *et al*.^12^	Italy	Biliary (36%)
Ahmed Ali *et al*.^13^	The Netherlands	Ethanol (23%)
Pelli *et al*.^14^	Finland	Ethanol (51%)
This study	India	Idiopathic (35%)

Four patients had died by the end of follow‐up. A 75‐year‐old female died from pancreatic carcinoma, 14 months after the index AP. Two patients died from a cerebrovascular accident and myocardial infarction, respectively, around 36 and 30 months, respectively, after index AP. Another 70‐year‐old male died from complications of an “abdominal tumor” 24 months after index AP.

Twenty‐eight patients either had RAP or had developed CP at the end of 5 years of follow‐up. Of these 28, 15 patients developed RAP (median 2, range 1–5) during 5 years of follow‐up. The index episode was idiopathic in nine, biliary in three, alcohol‐related in one, ascariasis‐related in one, and post‐ERCP in one, respectively. Their mean (± SEM) age was 27.4 (± 4.1) years and nine were male. On univariate analysis, young age was the only risk factor for the development of RAP (*P* < 0.0001). Thus, 12/15 (80.0%) of them were aged ≤ 35 years at the time of index AP vis‐à‐vis 26/72 (36.1%) of those who had remained asymptomatic. The etiology or severity of index AP was not a risk factor for recurrence.

The remaining 13 patients were diagnosed to have CP. Of these, nine had alcohol, three had biliary, and one was idiopathic as a cause for index attack of AP. A total of 42.8% of patients of alcoholic etiology went on to develop CP. Compared to the remaining 72 patients who neither developed RAP and/or CP, they were almost exclusively male (12/13 *vs* 43/72; *P* = 0.025) (Table 2), had alcohol as the etiology of index AP (9/13 *vs* 11/72; *P* < 0.0001), and more likely to have a moderate‐to‐severe index AP (10/13 *vs* 25/72; *P* = 0.003). They more frequently required interventions for their pseudocyst/WOPN during their index AP (5/13 *vs* 8/72; *P* = 0.025) and were more likely to have developed new‐onset diabetes at the end of 5 years (7/13 *vs* 9/71; *P* = 0.002).

**Table 2:** Univariate analysis

	RAP or CP, *N* = 28	Did not develop RAP/CP, *N* = 72	*P* value
Age (mean ± SEM) (year)	35.4 ± 3.1	44.3 ± 1.9	0.025
Age ≤ 35 years (%)	53.6	36.1	0.120
Male (%)	75.0	59.1	0.172
No comorbid illness (%)	71.4	72.2	0.940
Etiology (%)			0.127
Alcohol	35.7	15.3	
Biliary	21.4	34.7	
Idiopathic	35.7	34.7	
Acute necrotizing pancreatitis (%)	64.3	77.1	0.213
Severity (ATLANTA) (%)			0.037
Mild	42.9	65.3	
Moderate	28.6	20.8	
Severe	28.6	13.9	
Organ failure present (%)	28.6	14.3	0.145
Developed pseudocyst/WOPN (%)	58.3	42.1	0.226
Spontaneous resolution of pseudocyst/WOPN (%)	70.8	86.0	0.126

Of the six patients who had diabetes during index AP, two became normoglycemic at the end of 5 years. In addition, 17 patients developed new‐onset diabetes; 7 of them had also developed concomitant alcohol‐related CP. In the remaining 10 individuals, the index AP was idiopathic in 5, biliary in 4, and alcohol‐related in 1 patient.

On univariate regression analysis, it was found that those who developed new‐onset diabetes were more likely to be older at the time of index AP (mean [± SEM] age 48.5 [± 2.9] *vs* 40.0 [± 1.9]; *P* = 0.032), have had an alcoholic etiology (8/13 *vs* 13/82; *P* = 0.011), were more likely to have a necrotizing (16/17 *vs* 55/80; *P* = 0.036) and moderate‐to‐severe (13/17 *vs* 27/82; *P* = 0.001) index AP, have had more chance to developed organ failure (7/16 *vs* 9/74; *P* = 0.007), and less likely to have undergone spontaneous resolution of their pseudocysts/WOPN (9/16 *vs* 56/64; *P* = 0.009) during their index episode of AP.

## Discussion

A total of 122 admitted patients with AP were recruited in the index study of which 100 patients completed the follow‐up till 20–18 October. During follow–up, 15 patients developed RAP with younger age as the risk factor for recurrence. In addition, 13 patients developed transition to CP, with male sex and alcoholic etiology being risk factors for this outcome. At end of the follow‐up, a total of 17 patients went on to develop endocrine insufficiency in form of diabetes mellitus, 7 of whom had also developed concomitant CP.

Fifteen patients (15%) had a recurrent attack of pancreatitis over the follow‐up period. When these patients were compared with those who did not have any recurrence or chronicity (72/100) (Table 2), univariate analysis showed only young age (< 35 years) is a statistically significant risk of recurrence. The study by Lankisch^3^ showed a similar rate (17%) for recurrence and young age (< 40 years) as a significant risk factor for recurrence.

CP developed in 13 patients (13%). When compared to patients who did not have recurrence or chronicity (72/100), univariate analysis showed male sex, alcohol as etiology, and moderate to severe index attack as the significant risk factor for progression to chronicity. There have been several studies on long‐term follow‐up of AP reporting a progression rate to CP ranging from 4% to 24%^3^, ^14^, ^15^, ^16^ (Table 3). The wide range is due to the heterogeneity in nature of studies with differences in the rate of loss to follow‐up ranging up to 60% in some studies.^18^ The etiologies of index episode AP of the previous studies^3^, ^12^, ^13^, ^14^ also varies and may contribute to variable numbers of patients with CP (Table 1). The criteria for diagnosis of CP has also been variable, two large population‐based studies^15^, ^16^ used hospital registration data, whereas another large study^3^ used a self‐formulated score. In this study, the M‐ANNHEIM criteria^11^ was used to diagnose CP, which is a well‐accepted criterion based on multiple risk factors well suited for this purpose.

**Table 3:** Development of RAP and CP

	RAP	CP
Lankisch *et al*.^3^	17%	4%
Sarles *et al*.^17^		4%
Ahmed Ali *et al*.^13^	17%	7.6%
This study	15%	13%

In our study, 73.5% of patients with the initial attack had necrotizing pancreatitis (Table 4). This is like the study of Angelini *et al*.^12^ where 70.3% of patients with the initial attack had pancreatic necrosis. Both the results are in contradiction to other studies,^10^, ^19^ which showed only 10–20% of patients with the initial attack have pancreatic necrosis. The bias could be explained by the fact that both the studies were carried out in a tertiary referral center with the most severe cases being referred to these centers. Kloppel and Maillet^20^ had proposed the “necrosis fibrosis hypothesis” to explain the progression from AP to CP. Several studies^13^, ^21^ have showed that an increase chance of development of CP in patients of acute necrotizing pancreatitis supported this hypothesis. In this study also, a diagnosis of CP was more likely in those with moderate to severe index AP and these patients more frequently had interventions for pseudocysts or WOPN during index episodes.

**Table 4:** Necrotizing pancreatitis in initial attack

Study	Necrotizing pancreatitis (%)
Angelini *et al*.^12^	70.3
Beger *et al*.^19^	22.3
Pelli *et al*.^14^	22
Ahmed Ali *et al*.^13^	18.0
This study	73.5

Prior studies have shown that the rate of development of diabetes after AP is nearly 20–25%.^1^, ^18^ In this study, the chance of the development of diabetes after AP is 17%. Pancreatic necrosis leading to beta‐cell loss has been suggested as a pathophysiological basis for the development of diabetes after AP.^22^ This theory is supported by studies showing a higher frequency of diabetes in patients with pancreatic necrosis.^23^ Similar findings are reflected in our study with 22% of necrotizing pancreatitis patients developing diabetes over follow‐up. Apart from necrosis, older age and alcohol as etiology are other risk factors for the development of diabetes in our study.

One patient died due to pancreatic adenocarcinoma within a follow‐up duration of 75 months (absolute risk 1%). Similar findings have been noted in the study by Lankisch *et al*.^3^ where 0.8% of patients had died due to pancreatic cancer over a follow‐up period of 7.8 years. A population‐based cohort study from Denmark^24^ had shown that the risk of a diagnosis of pancreatic cancer remains after an episode of AP and persists for more than 5 years after index attack (absolute 5 years risk 0.87%).

## Conclusion

This study evaluates the natural history of AP. During follow‐up period, 15% of patients developed AP with young age (< 35 years) being the most significant risk factor for recurrence. A total of 13% of patients developed CP during follow‐up with male sex, alcohol as etiology, and moderate to severe initial attacks being the significant predictors for CP. A total of 17% of patients developed diabetes with alcoholic etiology and necrotizing pancreatitis among others, being significant predictors for the development of diabetes. A total of four patients died during the follow‐up period with one out of four deaths being due to pancreatic adenocarcinoma.

## Limitations

1. This was a single‐center study with the center being tertiary care so there is an inherent selection bias to this study whereby a majority of cases were moderate to severe cases of pancreatitis. This could have led to bias in the outcomes as many of the mild cases were missed.
2. This is a retrospective cohort study, so there is a risk of recall biases.
3. Endoscopic ultrasound was not routinely used to work up the etiology or at the end of follow‐up. This could have led to the missing of microliths in a number of cases, which could have caused AP, thereby increasing the number of idiopathic cases. Lack of endoscopic ultrasound study could have led to the missing of early CP in patients of RAP.
4. The effect of smoking as an etiology or determinant of severity was not studied. Smoking may be an independent risk factor for causing as well determining the severity of AP.
